# MicroRNAs as key regulators of cancer drug resistance: insights and future directions in chemotherapy, targeted-therapy, radiotherapy, and immunotherapy

**DOI:** 10.20517/cdr.2025.146

**Published:** 2025-12-10

**Authors:** Michael Attathikhun, Ancuta Jurj, George A. Calin

**Affiliations:** ^1^Department of Translational Molecular Pathology, The University of Texas MD Anderson Cancer Center, Houston, TX 77030, USA.; ^2^Center for RNA Interference and Non-Coding RNAs, The University of Texas MD Anderson Cancer Center, Houston, TX 77030, USA.; ^3^Cancer Biology, The University of Texas MD Anderson Cancer Center, Houston, TX 77030, USA.

**Keywords:** Non-coding RNAs, microRNAs, cancer, therapy, drug resistance

## Abstract

Cancer therapy remains an active field of investigation, particularly in understanding and overcoming therapy resistance. Small non-coding RNAs, such as microRNAs (miRNAs), are emerging as key regulators of cancer survival, progression, proliferation, invasion, migration, and metastasis. Although many studies have linked miRNAs to cancer therapy outcomes, significant questions remain regarding their precise molecular and cellular roles in therapy resistance. Increasing evidence shows that miRNAs influence critical pathways such as apoptosis, immune evasion, and other signaling cascades. However, there have been many setbacks because of the limitations in knowledge of each specific miRNA’s function. A deeper understanding of miRNA expression and function may enhance the development of more effective cancer therapeutics and improve overall survival of patients. This review explores the role of miRNA expression as a key regulator of therapeutic resistance in cancer patients.

## INTRODUCTION - MICRORNAS IN CANCER

According to data collected in the United States, the estimated number of cancer diagnoses in 2024 was approximately 2,001,140, with an extended projection of 611,720 cancer-related deaths. In the past few decades ranging from 1991 to 2021 there has been a significant decline in cancer-related mortality, largely due to advancements in therapeutic approaches and fundamental discoveries in cancer biology^[1]^. Despite these gains, the global cancer burden remains substantial, with projections estimating 28 million new cancer cases and 16.2 million cancer-related deaths by 2040, highlighting the increasing integration of oncology into modern healthcare system^[2]^. The global burden of cancer underscores the urgent need for targeted therapeutic strategies. MicroRNA (miRNA)-based therapeutics offer a unique opportunity to modulate gene expression with high specificity, including in therapy-resistant malignancies. As our understanding of miRNA biology in tumorigenesis deepens, their translation into clinical practice will be pivotal for achieving durable patient benefits. Importantly, overcoming challenges related to delivery, safety, and context-dependent activity remains essential for unlocking the full therapeutic potential of miRNAs.

Among the diverse factors influencing cancer initiation and progression, non-coding RNAs (ncRNAs) have emerged as critical regulatory molecules. These functional RNA transcripts lack protein-coding capacity but exert essential roles in gene expression control and tumorigenesis^[3]^. The most extensively studied classes include long non-coding RNAs (lncRNAs), miRNAs, and circular RNAs (circRNAs). LncRNAs, defined by a length exceeding 200 nucleotides, can act as oncogenes or tumor suppressors depending on their expression patterns and molecular interactions^[4,5]^. CircRNAs, first described in 1970 by Frederick Sanger *et al.* as covalently closed single-stranded RNA loops^[6]^, exhibit remarkable stability due to resistance to exonucleolytic degradation at their termini. They are further distinguished by evolutionary conservation, tissue-specific expression, and predominantly cytoplasmic localization^[7]^. Moreover, RNA modifications - including alternative splicing, RNA editing, methylation, and pseudouridylation - expand the regulatory repertoire of ncRNAs, contributing to their context-dependent roles in cancer biology^[3]^.

Among ncRNAs, miRNAs - small RNAs of ~19-24 nucleotides - have emerged as central regulators of oncogenesis and tumor suppression. By binding to complementary sequences within target messenger RNAs (mRNAs), miRNAs mediate translational repression or mRNA degradation^[8]^, thereby exerting post-transcriptional control over a wide spectrum of cellular processes, including proliferation, apoptosis, immune evasion, and metastasis^[9]^. MiRNAs have consistently remained at the forefront of ncRNA research. Their discovery, recognized by the 2024 Nobel Prize in Physiology or Medicine awarded to Gary Ruvkun and Victor Ambros, stemmed from pioneering studies in *C. elegans*, where lin-4 was shown to regulate *lin-14* expression^[10]^. The subsequent identification of let-7 established miRNAs as evolutionarily conserved regulators of developmental timing and cellular homeostasis^[10]^. Building on these insights, miRNA-based therapeutics are now being explored across diverse diseases - including cancer, fibrosis, and viral infections - with miRNA mimics and inhibitors entering clinical trials to restore or antagonize dysregulated pathways, offering novel and precise treatment strategies^[11]^. Notably, miRNAs’ role in modulating immune evasion is through the regulation of immune checkpoint (IC) expression, antigen presentation, and inflammatory signaling pathways to enhance the target of immune-mediated clearance of diseased cells^[12]^.

MiRNAs have become increasingly valuable in oncology research, both as diagnostic biomarkers and therapeutic targets. Specific miRNAs, such as miR-10b and miR-196a in glioblastoma, serve as circulating biomarkers, as their elevated expression correlates with poor patient survival, underscoring their potential as valuable biomarkers for early detection and prognostic assessment^[13]^. MiRNAs bind to complementary sequences on target mRNAs, thereby disrupting the translational mechanism and inhibiting protein production^[8]^. These post-transcriptional regulators play key roles in gene expression control. Importantly, miRNAs display characteristics that function as either tumor suppressors or oncogenic promoters [oncogenic miRNAs (oncomiRs)], depending on their target genes and expression context^[14]^. Tumor-suppressive miRNAs are typically downregulated in cancer and can inhibit tumor growth and proliferation by promoting cell cycle arrest and apoptosis. However, miR-371/373 and miR-302/367 clusters in germ cell tumors (GCTs) contribute to oncogenesis and their overexpression further drives uncontrolled cell proliferation^[14,15]^. Many miRNAs act as oncogenes when overexpressed or hyperactivated during tumor development^[9]^. It was proved that miRNAs, such as miR-17-92 cluster (miRs-17, -18a, -19a, -20a, -19b, and -92a) promotes tumor growth by targeting tumor suppressor genes such as phosphatase and tensin homolog deleted on chromosome 10 (PTEN) leading to a negative correlation with tumor-suppressive miRNAs as shown with miR-15a and miR-16-1 in chronic lymphocytic lymphoma^[8,16]^. Elevated expression of these oncomiRs is associated with enhanced tumor progression, metastasis, and poor clinical outcomes.

**Figure 1:**
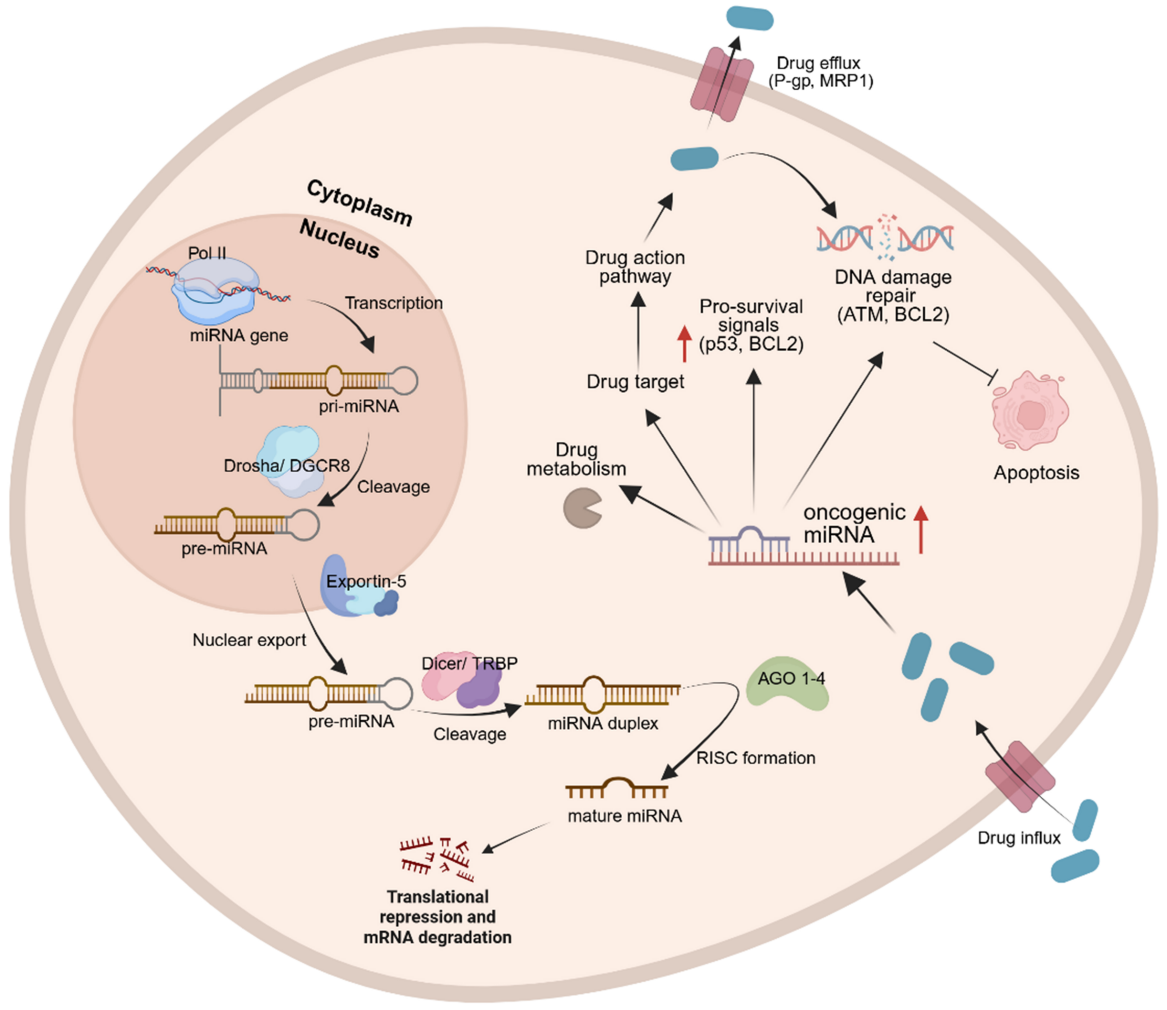
Biogenesis of miRNAs begins with pri-miRNA transcription, which interact with DGCR8 and DROSHA. Guided by DGCR8, DROSHA cleaves the stem-loop structure from pri-miRNAs in the nucleus, generating pre-miRNAs. Pre-miRNAs are then exported to the cytoplasm via exportin-5, where DICER further cleaves them to form mature miRNA complexes. In the presence of a stimulus, such as a drug, enzymatic modifications can occur to activate or inactivate the drug. MiRNAs can influence drug responses by promoting drug efflux from the cytoplasm before the full therapeutic dosage is delivered or by modulating DDR mechanisms. Created in BioRender. Jurj, M. (2025) https://BioRender.com/s6bhh3f. MiRNA: MicroRNA; pri-miRNAs: primary miRNAs; DGCR8: DiGeorge syndrome critical region 8; pre-miRNAs: precursor miRNAs; DDR: DNA damage responses; TRBP: HIV-1 TAR RNA-binding protein; AGO: Argonaute; RISC: RNA-induced silencing complex; P-gp: P-glycoprotein; MRP1: MDR-associated protein 1; ATM: ataxia telangiectasia mutated; BCL2: B-cell lymphoma 2.

Biogenesis of miRNAs begins with the transcription of primary miRNA (pri-miRNA) transcripts, which are processed in the nucleus by DiGeorge syndrome critical region 8 (DGCR8) and the ribonuclease enzyme, DROSHA, guided by DGCR8 to cleave the stem-loop structure from pri-miRNAs, generating precursor miRNA (pre-miRNA) transcripts. These pre-miRNAs are subsequently exported to the cytoplasm by Exportin-5, where they undergo further cleavage by the ribonuclease DICER to generate ~22-nucleotide duplexes. The guide strand is then incorporated into Argonaute (AGO) proteins within the RNA-induced silencing complex (RISC), enabling post-transcriptional regulation of target RNAs [Figure 1]^[17]^. Beyond canonical gene silencing, miRNAs display functional versatility, including regulation of nuclear ncRNAs and, in certain contexts, the translational activation of mRNAs^[17]^.

In this review, we focus on the importance of ncRNAs, particularly miRNAs, in mediating cancer therapy resistance - including resistance to chemotherapy, targeted therapy, radiotherapy (RT), and IC inhibitors (ICIs). By dissecting the tumor-suppressive and oncogenic functions of specific miRNAs, we highlight their mechanistic contributions to therapy resistance and identify opportunities for improving treatment efficacy. Notably, many miRNAs exhibit context-dependent functions: for instance, miR-125b induces apoptosis and suppresses tumor growth in breast cancer yet promotes proliferation and chemoresistance in leukemia^[18]^. Similarly, miR-9 acts as a metastatic suppressor in neuroblastoma but enhances invasion and metastasis in breast cancer through repression of E-cadherin^[19]^. Other dual-role miRNAs include miR-21, which promotes oncogenesis in solid tumors through PTEN and programmed cell death protein 4 (PDCD4) suppression^[20]^ but displays tumor-suppressive activity in certain leukemias via modulation of apoptosis pathways^[21]^; miR-155, a well-established oncogene in lymphoma, yet protective in inflammatory breast cancer through immune modulation^[22]^; and the let-7 family, generally tumor-suppressive, but in some contexts [e.g., Kirsten rat sarcoma viral oncogene homolog (KRAS)-mutant lung cancer] paradoxically enhances tumor progression by stabilizing oncogenic signaling networks^[23]^.

This functional plasticity complicates the therapeutic translation of miRNAs but also underscores their unique value as biomarkers - where expression patterns and regulatory context can provide insight into tumor type, stage, and potential treatment response.

## RESISTANCE OF THERAPY MEDIATED THROUGH MIRNAS

**Figure 2:**
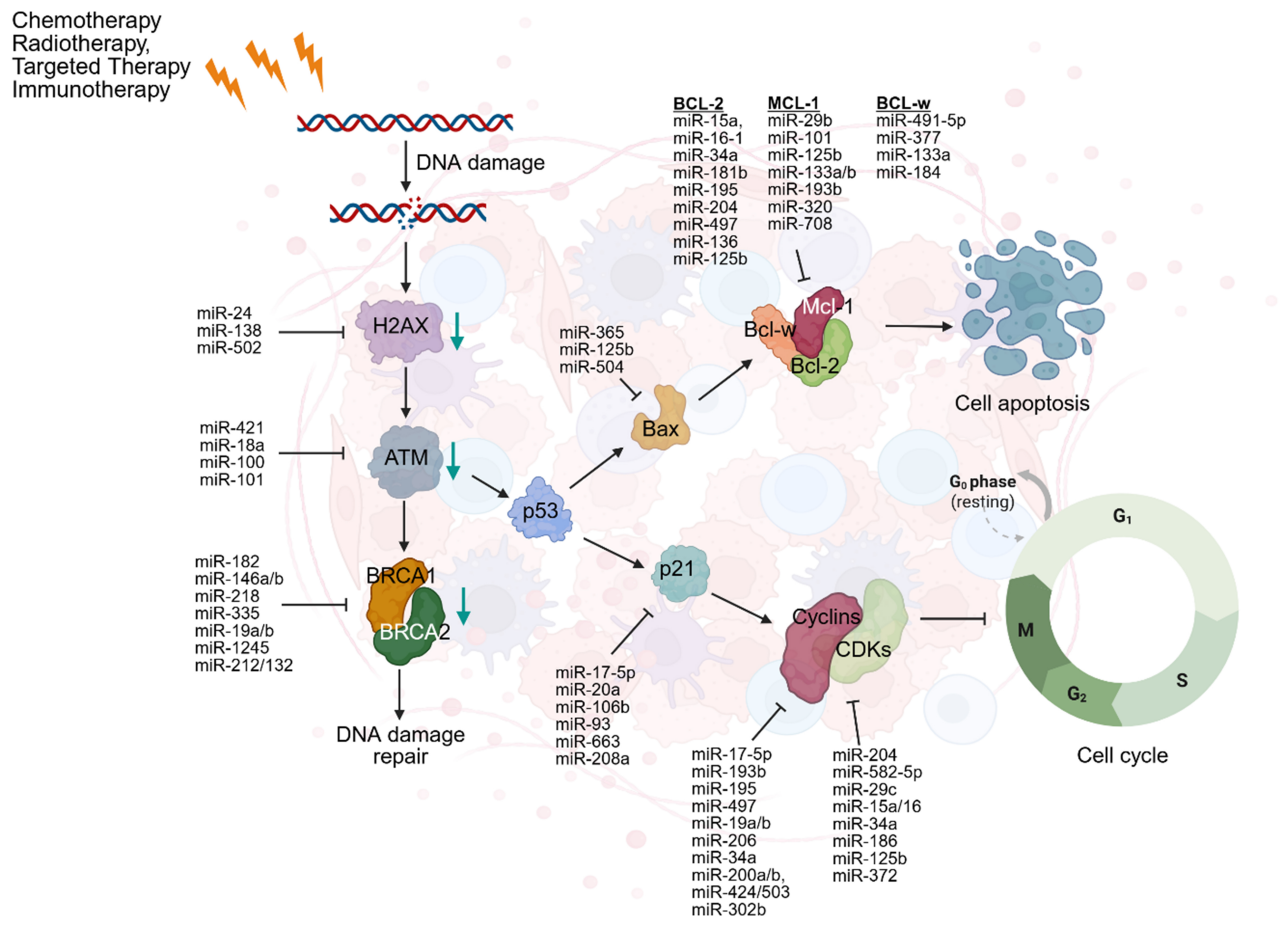
DDR is a cellular mechanism that detects and repairs DNA damage including SSBs and DSBs, caused by treatments. DNA damage activates H2AX which recruits ATM to initiate the DDR. ATM activates BRCA1/2 for repair, while p53 drives apoptosis via BAX by inhibiting Bcl-2, Bcl-w, and Mcl-1, leading to cell death. p53 can also transcribe p21 which inhibits the CDK complexes, promoting cell cycle progression. Created in BioRender. Attathikhun, M. (2025) https://BioRender.com/cjd64fm. DDR: DNA damage responses; SSBs: single-stranded breaks; DSBs: double-stranded breaks; H2AX: H2A histone family member X; ATM: ataxia telangiectasia mutated; BRCA1/2: breast cancer type 1/2 early onset; BAX: BCL2-associated X protein; CDK: cyclin-dependent kinase.

Many mechanistic pathways involving ncRNAs contribute to both intrinsic and acquired forms of therapy resistance. These pathways can promote oncogene modification, increased survivability, and facilitate evolutionary adaptations, further increasing the plasticity of cancerous cells^[17]^. This plasticity is reflected in various changes, one of which is the phenotypic transition of cancerous cells from an epithelial to a mesenchymal state or its reversal. Synergistically, the increased development and plasticity of malignant cells initiate resistance-related traits. Consequently, resistance is intrinsically variable, influenced by pre-existing mechanisms that can induce biological challenges coupled with ncRNAs. Resistance can be classified as either acquired or intrinsic, depending on its timing and origin. Intrinsic resistance arises from pre-existing features of the tumor, while acquired resistance develops in response to therapeutic pressure^[24]^. Notably, miRNAs play a central role in resistance, particularly through their involvement in DNA damage responses (DDR). Although limited tumor therapy resistance is driven by acquired mechanisms, a stronger correlation often exists between intrinsic tumor sensitivity and therapeutic resistance^[25]^. Many mechanisms of resistance include hypoxia, dysregulation of the cell cycle, inhibition of apoptosis, and, notably, aberrant DDR pathways. DNA damage is classified as single-stranded breaks (SSBs) or double-stranded breaks (DSBs). These abnormalities of DDR are caused by the direct binding of treatments, such as chemotherapy, which directly interact with DNA structure and cause structural degradation^[26]^. These disruptions alter the tumor microenvironment and reduce the effectiveness of treatments, while simultaneously serving as a direct mechanism to promote tumor survival. MiRNAs are intimately involved in DDR regulation by modulating the expression of oncogenes involved in repair pathways, and cell cycle regulators. Their dysregulated expression, either upregulation or downregulation, has been increasingly linked to the development of drug resistance. Ultimately, understanding the role of miRNAs in these pathways offers potential for improving therapeutic outcomes and overcoming resistance [Figure 2]^[27]^.

Several mechanistic examples underscore this role. Overexpression of miR-155 promotes genomic instability and chemotherapy resistance by impairing high-fidelity DNA repair and favoring error-prone pathways such as non-homologous end joining^[28]^. In this case, causality has been established, since the knockdown of miR-155 reverses resistance phenotypes. Likewise, miR-21 confers resistance by suppressing tumor suppressors including PTEN and tissue inhibitor of metalloproteinase 3 (TIMP3), thereby attenuating apoptosis and promoting survival under therapeutic stress^[29,30]^; functional rescue experiments here also support a causal role. Conversely, tumor-suppressive miRNAs can enhance sensitivity to DNA-damaging therapies. For example, restoration of miR-34a expression, frequently lost in many cancers, sensitizes tumor cells to RT and chemotherapy by targeting DDR components and promoting apoptosis^[31]^. Similarly, miR-200c has been implicated in reversing epithelial-mesenchymal transition (EMT)-associated drug resistance, restoring responsiveness to targeted therapies^[32]^. However, not all reported associations are causal: in several cases, miRNA expression profiles correlate with resistance phenotypes without definitive functional validation. Thus, while certain miRNAs have clear mechanistic roles in resistance, many others remain correlational biomarkers whose causal contribution to therapy resistance still needs rigorous testing. Together, these findings emphasize that miRNAs can function as both mediators of resistance and sensitizers to therapy, depending on their expression patterns and regulatory targets. This duality not only complicates therapeutic strategies but also highlights the need for precision approaches that integrate functional validation with biomarker discovery.

### Resistance to chemotherapy

Chemotherapy remains a cornerstone in the treatment of rapidly dividing cancer cells, aiming to reduce tumor burden and control cancer progression^[33]^. However, its efficacy is frequently limited by significant side effects, including cytopenia and immune suppression, as well as the emergence of resistance^[33]^. Despite this dual effect, chemotherapy remains a preferred treatment option for various types of cancer. There are many different classes of chemotherapeutic agents including platinum-based compounds, carboplatin, cisplatin, nedaplatin, as well as alkylating agents and antimetabolites such as methotrexate, cytarabine, and thioguanine^[2]^. The primary mechanism of action involves inhibiting cancer cell proliferation and metastasis by damaging DNA and preventing its replication. However, it has been discovered that miRNAs can disrupt these processes and contribute to resistance to various chemotherapy subtypes [Table 1].

**Table 1:** MiRNAs’ role in resistance to cancer therapeutics - chemotherapy

**ncRNA**	**Cancer drug**	**Expression of miRNA**	**Cancer/cell type**	**Resistance pathway**	**Ref.**
**Chemotherapy**					
miR-371a-3p	Carboplatin, Cisplatin, Etoposide	Upregulated	Testicular GCTs	hCGt and LDH levels affected	[9]
miR-144	Cisplatin	Downregulated	Cervical cancer	LHX2 overexpression	[34]
miR-363	Cisplatin	Downregulated	Ovarian cancer	SNAIL overexpression	[35]
miR-769-5p	Cisplatin	Upregulated	Gastric cancer	Cas-9 deactivation	[36]
miR-19	Taxol (Paclitaxel)	Upregulated	Breast cancer	Targets PTEN	[37]
miR-34a	Docetaxel	Downregulated	Breast cancer	Inhibits tumor suppressant mRNA	[38]
miR-21	Docetaxel	Upregulated	Breast cancer	VEGF overexpression	[39]
miR-449	Doxorubicin	Downregulated	TNBC	SIRT1-HDAC1 negative feedback loop	[40]
miR-125b-5p, miR-146a-5p, miR-484, miR-1246-5p, miR-1260b	Neoadjuvant chemotherapy	Upregulated	Breast cancer	Affected genes *BAK1*, *NOVA1*, *PTGER4*, *RTKN2*, *AGO1*, *CAP1*	[41]
miR-17-5p	Cisplatin	Upregulated	Gastric cancer	Overexpression of E2F, p21, and upregulation of MCL1	[42]

MiRNAs: MicroRNAs; ncRNA: non-coding RNA; GCTs: germ cell tumors; hCGt: human chorionic gonadotropin; LDH: lactate dehydrogenase; LHX2: lim homeobox transcription factor 2; Cas-9: caspase 9; PTEN: phosphatase and tensin homolog; mRNA: messenger RNA; VEGF: vascular endothelial growth factor; TNBC: triple-negative breast cancer; SIRT1: sirtuin 1; HDAC1: histone deacetylase 1; BAK1: BCL2 antagonist/killer 1; NOVA1: neuro oncological ventral antigen 1; PTGER4: prostaglandin E receptor 4; RTKN2: rho-associated coiled-coil containing protein 2; AGO1: argonaute RISC catalytic component 1; CAP1: carcinoembryonic antigen peptide-1; MCL1: myeloid cell leukemia sequence 1.

Recent findings show that dysregulated miRNA expression can affect drug sensitivity. The surge in identified miRNAs - for example, miR-144, which is downregulated in cervical cancer; miR-363, in ovarian cancer; and miR-769-5p, which is upregulated in gastric cancer - demonstrates their effects on anti-cancer drugs such as cisplatin^[34-36]^. For instance, the upregulation of miR-769-5p in cisplatin-treated gastric cancer cells promotes deactivation of caspase-9 (Cas-9) in response to exosome stimuli^[36]^. Therefore, Cas-9 deactivation leads to suppression of p53 activity through E3 ubiquitin ligase-mediated degradation, contributing to resistance against cisplatin and other DNA-damaging agents. This clinical relevance was supported by data from 75 gastric cancer patients, in whom miR-769-5p was significantly overexpressed in tumor tissue compared to normal tissue and was correlated with poor prognosis^[36]^. Cisplatin, one of the most commonly used chemotherapy drugs, is a platinum-based compound. The mechanism of action involves a cytotoxic effect induced when a chloride ion detaches from cisplatin, allowing the polarity of the negatively charged DNA strand to bind to the positively charged platinum-based drug. Consequently, this binding disrupts DNA structure, inhibiting DNA replication and cell proliferation^[43]^.

The duration and intensity of chemotherapy exposure influence the development of chemoresistance, whether single-drug or multidrug resistance (MDR). MiRNAs play a crucial regulatory role in this process. For instance, in breast cancer, miR-19 and miR-21 are overexpressed seven-fold in chemo-resistant MCF7 cell lines compared to MCF10a non-tumor cells, emphasizing an adaptive response of cancer cells^[39]^. These miRNAs may promote changes in cell phenotype, enhancing migration, adhesion, and proliferation. Conversely, miR-298 targets the multidrug resistance 1 (*MDR-1*) gene, a specific glycoprotein classified as P-glycoprotein (P-gp), a membrane transporter that expels chemotherapeutic drugs from the cell. In metastatic breast cancer MDA-MB-231, downregulation of miR-298 results in increased P-gp expression, leading to chemoresistance^[37,44]^.

Doxorubicin, a key chemotherapeutic agent used in the treatment of triple-negative breast cancer (TNBC), functions as a DNA topoisomerase II inhibitor. It exerts its effects through multiple mechanisms, including inhibition of DNA repair, induction of cell death, and generation of oxidative stress on cellular membranes^[45]^. The miR-449 family, comprising miR-449a, miR-449b-5p, and miR-449c-5p, is downregulated in response to doxorubicin, a process driven by histone deacetylation. The removal of acetyl groups from histones promotes gene silencing, and this response is mediated in part by upregulation of histone deacetylase 1 (HDAC1) and sirtuin 1 (SIRT1) following doxorubicin treatment in TNBC. Silencing HDAC1 and SIRT1 restores miR-449 expression, thereby sensitizing cancer cells to doxorubicin^[40]^.

Therapeutically, HDAC1 and SIRT1 can be targeted using small-molecule inhibitors that disrupt their enzymatic activity, reversing the epigenetic repression of tumor-suppressive miRNAs, including the miR-449 family. HDAC inhibitors (HDACis) such as Vorinostat, Belinostat, Romidepsin, and Panobinostat have been developed to modulate histone acetylation and deacetylation, restoring transcriptional balance in cancer cells. In preclinical and early clinical studies, Vorinostat in combination with Decitabine, a DNA methyltransferase inhibitor (DNMTi), has been evaluated in 43 patients with non-Hodgkin lymphoma (NHL) and advanced solid tumors (ASTs). Nine dose levels with sequential drug administration were assessed in Phase 1 studies, followed by a Phase 2 schedule in which Vorinostat was administered at 200 mg twice daily on days six and twelve, while Decitabine was given at 10 mg/m^2^/day from days one to five. This combination was well tolerated across schedules and demonstrated stabilization of diverse tumor types^[46]^. SIRT1 inhibitors, such as the small molecule EX-527, inhibit the NAD^+^-dependent deacetylase activity of SIRT1, restoring both gene expression and histone acetylation^[47]^. EX-527 has shown the highest efficacy in models with upregulated SIRT1 activity, as demonstrated in hepatocellular carcinoma (HCC) cell lines HepG2 and Huh7. Pharmacological inhibition of SIRT1 with EX-527 results in downregulation of P-gp and MDR-associated protein 3 (MRP3), highlighting an additional mechanism by which SIRT1 contributes to MDR^[48,49]^. Collectively, these findings underscore the therapeutic relevance of targeting HDAC1 and SIRT1 to restore tumor-suppressive miRNA expression and enhance chemosensitivity, providing a mechanistic basis for overcoming resistance in TNBC and other malignancies.

Overall, miRNAs contribute to drug resistance through multiple mechanisms, including modulation of intracellular drug concentration, alteration of drug targets, suppression of apoptosis, and regulation of DDR pathways^[37,50]^. Their effects are highly context-dependent, varying with tissue type, tumor state, and chemotherapeutic agent^[51]^. Consequently, dissecting the causal *vs.* correlational roles of miRNAs in chemoresistance is critical for translating mechanistic insights into personalized therapeutic interventions.

### Resistance to targeted therapy

A more selective intervention in oncology involves targeted therapies, such as small-molecule inhibitors. Kinase inhibitors such as imatinib, or multi-kinase inhibitors such as sorafenib, selectively target specific molecular pathways or proteins involved in tumor development^[52]^. These therapies offer the advantage of precise targeting and reduced off-target toxicity compared to chemotherapy. However, their limitations include a narrow spectrum of action, effectiveness only in cancers with specific molecular aberrations, and the emergence of drug resistance, which is increasingly associated with miRNA expression among other factors^[52]^.

In non-small cell lung cancer (NSCLC), chemotherapy drugs such as cisplatin, when combined with targeted agents, have demonstrated improved outcomes^[53]^. Resistance to cisplatin is often associated with disruption of apoptotic pathways, and elevated miR-10b expression contributes to this resistance by inhibiting p53 signaling^[53]^. NSCLC patients harboring epidermal growth factor receptor (EGFR) mutations may benefit from tyrosine kinase inhibitors (TKIs); however, EMT can promote TKI resistance through interference with apoptosis^[53]^. In this context, miRNA expression patterns could serve as predictive biomarkers of TKI responsiveness, guiding patient stratification, and personalized treatment decisions.

Sorafenib, a multi-target TKI, is widely used to inhibit proliferative activity in late-stage HCC^[45]^. It primarily targets the Ras/Raf/mitogen-activated protein kinase/extracellular signal-regulated kinase kinase (MEK)/extracellular signal-regulated kinase (ERK) signaling pathway by inhibiting Ras-1 and B-Raf. Ras-1 normally activates downstream signaling by binding guanosine triphosphate (GTP), which in turn stimulates B-Raf and the MEK/ERK pathway, promoting cancer cell differentiation, proliferation, and survival^[54]^. While TKIs such as sorafenib interfere with angiogenesis and proliferation pathways, only approximately 30% of HCC patients achieve a therapeutic benefit, and many develop resistance or experience side effects such as gastrointestinal toxicity^[54]^. The limited efficacy highlights the need for predictive biomarkers to guide therapy. Recent studies have identified miRNAs as potential biomarkers to stratify patients likely to respond to sorafenib. For instance, miR-486-3p is downregulated in sorafenib-resistant HCC cell lines such as SK-Hep-1-SR, HepG2-SR, and Huh7-SR, and targets key mediators such as EGFR and fibroblast growth factor receptor-4 (FGFR4)^[55]^. Clinically, measuring miR-486-3p levels in patient tumors or circulating exosomes could predict responsiveness to sorafenib, enabling more personalized therapy. Moreover, restoring miR-486-3p expression using miRNA mimics has been shown in preclinical models to sensitize resistant HCC cells to sorafenib, demonstrating a potential therapeutic strategy to overcome drug resistance. These findings underscore the dual utility of miRNAs in HCC: as predictive biomarkers for patient selection and as actionable targets to enhance TKI efficacy [Table 2].

**Table 2:** MiRNAs’ role in resistance to cancer therapeutics - targeted therapy

**ncRNA**	**Cancer drug**	**Expression of miRNA**	**Cancer/cell type**	**Resistance pathway**	**Ref.**
**Targeted therapy**					
miR-10b	Sorafenib	Upregulated	NSCLC	Inhibition of p53	[54]
miR-486-3p	Sorafenib	Downregulated	HCC	Targets EGFR and FGRF4	[55]
miR-200-b	Sorafenib	Downregulated	Breast cancer	Overexpression of c-MYK and ZEB1	[56]
miR-200-c	Sorafenib	Downregulated	Breast cancer	Overexpression of c-MYK and ZEB1	[56]
miR-4443, miR-4488, miR-204-5p, miR-199b-5p	BRAF inhibitors	miR-4443 and miR-4488 upregulated miR-204-5p and miR-199b-5p downregulated	Melanoma	Activation of the YAP/TAZ mechanotransduction pathway	[57]
miR-17-3p, miR-222, miR-340	Intraperitoneal delivery of mimic miRNA	Upregulation of miR-222 Downregulation of miR-17-3p and miR-340	Glioblastoma	Decrease in AKT signaling pathway	[58]
Let-7 family, miR-199a, miR-375	Cetuximab (EGFR inhibitor)	Downregulated let-7 Upregulation of miR-199a and miR-375	Gastrointestinal cancers	KRAS, AKT pathway	[59]
miR-223	Trastuzumab	Upregulated	HER2-positive gastric cancer	FBXW7 downregulation	[59]
miR-200c, miR-221, miR-222	Trastuzumab Gefitinib	Downregulated miR-200c Upregulation of miR-221 and miR-222	Breast cancer Lung cancer	TGFβ, mTOR, Wnt, MAPK signaling	[60]

MiRNAs: MicroRNAs; ncRNA: non-coding RNA; NSCLC: non-small cell lung cancer; HCC: hepatocellular carcinom; EGFR: epidermal growth factor receptor; FGRF4: fibroblast growth factor receptor 4; c-MYK: cellular Myc; ZEB1: zinc-finger E-box binding homeobox 1; BRAF: B-Raf proto-oncogene, serine/threonine kinase; YAP/TAZ: yes-associated protein/transcriptional co-activator with PDZ-binding motif; AKT: protein kinase B (PKB); KRAS: Kirsten rat sarcoma viral oncogene homolog; HER2: human epidermal growth receptor 2; FBXW7: F-box and WD repeat domain containing 7; TGFβ: transforming growth factor beta; mTOR: mammalian target of rapamycin; Wnt: wingless-related integration site; MAPK: mitogen-activated protein kinase.

Similarly, the expression of miR-200b/c has been linked to treatment sensitivity in estrogen receptor-positive (ER+) breast cancer cells. In tamoxifen-resistant MCF7 (TAM-MCF7) cells that are also resistant to sorafenib, researchers observed increased expression of c-MYC, decreased levels of miR-200b/c, and elevated expression of mesenchymal markers such as zinc-finger E-box binding homeobox 1 (ZEB1). This profile is associated with an aggressive phenotype and enhanced cancer stemness, contributing to disease progression. By contrast, standard MCF7 cells showed stable miR-200a/b/c expression, low mesenchymal marker expression, and reduced c-MYC levels^[56]^. Restoration of miR-200b/c expression via pre-miR transfections suppresses c-MYB and partially reverses EMT, highlighting miR-200b/c as a therapeutic target for overcoming resistance and as a biomarker for predicting treatment efficacy. Conversely, inhibition of miR-200b/c in standard MCF7 cells promotes EMT, demonstrating the predictive value of miRNA expression patterns in assessing therapeutic risk^[56]^.

The let-7 miRNA family provides a clinically relevant example of miRNA-mediated modulation of therapeutic response. Downregulation of let-7 in metastatic colorectal cancer has been linked to resistance to cetuximab, particularly in tumors harboring KRAS mutations and activated mitogen-activated protein kinase (MAPK) signaling^[59]^. Genetic variants in the KRAS 3′-untranslated region (UTR), such as the LCS6 (the 6th *let-7* complementary site in the *KRAS* 3′UTR) polymorphism, can impair let-7 binding, further reducing its inhibitory effect on KRAS expression. In a clinical cohort of 234 patients, 100 carried the wild-type LCS6 T/T genotype, while 34 harbored the LCS6 G variant allele. Analysis indicated that patients with KRAS mutations and the LCS6 variant had poorer responses, highlighting how genetic modulation of miRNA binding contributes to therapeutic resistance^[61]^. Modulating let-7 levels can help overcome resistance: preclinical studies suggest that restoring let-7 expression suppresses KRAS signaling, thereby sensitizing tumor cells to anti-EGFR therapies. Clinically, Angerilli *et al.* analyzed archived formaldehyde fixed-paraffin embedded (FFPE) tumor samples from patients receiving third-line cetuximab combined with irinotecan. They observed that higher let-7 expression correlated with improved overall survival and enhanced responsiveness to anti-EGFR therapy [Table 2]^[59]^. These findings illustrate that let-7 can function both as a predictive biomarker, guiding patient stratification and selection for targeted therapy, and as a therapeutic target, where its modulation may restore drug sensitivity and overcome resistance mechanisms. Integrating let-7 profiling into clinical workflows could therefore inform personalized treatment strategies and optimize therapeutic outcomes^[59]^.

### Resistance to RT

The concept of RT is more closely related to chemotherapy than to targeted therapy due to its nonspecific targeting of both cancerous and healthy cells. RT uses ionizing radiation (IR), typically in the form of external gamma-ray beams, to inhibit tumorigenesis and induce tumor shrinkage^[62]^. There are multiple types of beam radiation therapies, both internal and external. Internal applications include brachytherapy, while external beam techniques, such as three-dimensional conformal radiation therapy (3D-CRT), are more commonly used. One of the key advantages of RT is its ability to minimize damage to healthy tissues while remaining non-invasive. However, RT typically requires multiple sessions over several weeks, which increases the risk of secondary malignancies^[63]^.

Similar to chemotherapy, RT damages DNA to induce cancer cell death through multiple signaling pathways. Namely, reactive oxygen species (ROS) are generated during infrared (IR) exposure; these highly reactive molecules bind to and damage DNA, leading to SSB or DSB^[62]^. Activation of DDR pathways, however, can promote radioresistance, limiting therapeutic efficacy. MiRNAs are intimately involved in this process. For instance, ataxia telangiectasia mutated (ATM), a central DDR kinase, is activated by IR and phosphorylates downstream transcription factors that regulate miRNA expression^[64]^. ZEB1, a transcription factor stabilized by ATM-mediated phosphorylation, suppresses the expression of miR-205-5p in nasopharyngeal carcinoma (NPC)^[64,65]^. Mechanistically, it binds to the promoter region of the *MIR205* gene, recruiting co-repressors such as HDACs, leading to chromatin condensation and transcriptional silencing. Concurrently, ZEB1 interacts with CHK1, stabilizing this checkpoint kinase, which enhances the recruitment of DNA repair proteins to sites of DSBs and promotes cell cycle arrest to allow repair. MiR-205-5p normally inhibits both ZEB1 and components of the DNA repair machinery, including RAD17 and RAD51, which are essential for homologous recombination repair. Downregulation of miR-205-5p by ZEB1/CHK1-mediated repression thus derepresses DNA repair genes, increases repair efficiency, and contributes to radioresistance [Table 3]^[64,65]^. This axis - ATM → ZEB1/CHK1 → miR-205-5p → DNA repair - highlights a precise regulatory network whereby miRNAs integrate DDR signaling and transcriptional control to modulate therapeutic response. Targeting this pathway, either by restoring miR-205-5p expression or disrupting ZEB1/CHK1 activity, represents a potential strategy to overcome radioresistance and improve RT efficacy.

**Table 3:** MiRNAs’ role in resistance to cancer therapeutics - RT

**ncRNA**	**Cancer drug**	**Expression of miRNA**	**Cancer/cell type**	**Resistance pathway**	**Ref.**
**RT**					
miR-141, miR-375	Brachytherapy	Upregulated	Prostate cancer	PI3K/AKT/mTOR pathway	[9]
miR-34a	Ionized radiation	Upregulation	Prostate cancer	ATM pathway causes downregulation of PP1a gamma	[62]
miR-205-5p	γ-Ionized radiation	Upregulated	Breast cancer	Downregulation of ZEB1, CHK1, and ATM	[64,65]
miR-205	Ionized radiation	Upregulated	NPC	PTEN/AKT pathway	[66]
miR-216a	Ionized radiation	Downregulated	Human bronchial epithelial cells	Increase in Beclin-1	[67]
miR-21	Ionized radiation	Upregulated	CML and B-cell leukemias	Downregulation of PTEN and upregulation of VEGF through PI3K/AKT pathway	[67]
miR-214	Ionized radiation	Upregulated	NSCLC	Upregulation of p38MAPK pathway	[68]
miR-31-5p, miR-613, miR-340-3p	Ionized radiation	Downregulated	NPC	DDR and cell cycle regulation	[69]
miR-196a	Ionized radiation	Upregulated	Head and neck squamous cell carcinoma	ANXA1 inhibition, upregulation of EGFR, activation of PI3K/AKT and RAS pathway	[70]

MiRNAs: MicroRNAs; RT: radiotherapy; ncRNA: non-coding RNA; PI3K: phosphatidylinositol-3 kinase; AKT: protein kinase B (PKB); mTOR: mammalian target of rapamycin; ATM: ataxia telangiectasia mutated; PP1a gamma: protein phosphatase 1; ZEB1: zinc-finger E-box binding homeobox 1; CHK1: checkpoint kinase 1; NPC: nasopharyngeal carcinoma; PTEN: phosphatase and tensin homolog; CML: chronic myelogenous leukemia; VEGF: vascular endothelial growth factor; NSCLC: non-small cell lung cancer; p38MAPK: p38 mitogen-activated protein kinase; DDR: DNA damage responses; ANXA1: annexin A 1; EGFR: epidermal growth factor receptor; RAS: rat sarcoma virus.

Overall, miRNAs are recognized as key regulators of autophagy, a process frequently activated by RT. For instance, in human bronchial epithelial cells, IR-induced downregulation of miR-216a leads to increased beclin-1 expression, a central autophagy-related protein involved in cellular degradation pathways^[67]^. Therapeutically, restoring or fine-tuning miR-216a levels could modulate autophagic flux, enhancing radiosensitivity by preventing excessive autophagy that allows cancer cells to survive RT. Potential strategies include the use of miRNA mimics to restore miR-216a activity or small molecules that stabilize miR-216a expression^[71]^. RT protocols vary by timing and duration, and prolonged exposure is associated with radiation-induced carcinogenesis. IR can alter miRNA regulation, promoting cellular plasticity that enables cancer cells to adapt and survive. In chronic myelogenous leukemia (CML) and B-cell leukemias, miR-21 is upregulated in response to radiation and influences downstream targets such as PTEN, vascular endothelial growth factor (VEGF), and hypoxia-inducible factor-1α (HIF1-α)^[72]^. PTEN, a tumor suppressor, is downregulated, while VEGF is upregulated, promoting angiogenesis through the phosphatidylinositol-3 kinase (PI3K)/protein kinase B (PKB, AKT) pathway^[67]^. Furthermore, upregulation of HIF1-α promotes tumor survival by activating genes that help cells adapt to hypoxic conditions^[67]^. In gastric cancer cell lines MKN and AGS, transfection with anti-miR-21 restored the expression of tumor suppressors including C-C motif chemokine ligand 28 (CCL28), nuclear receptor subfamily 3 group C member 2 (NR3C2), and synaptopodin 2 (SNYP02), highlighting potential targets for overcoming radioresistance^[73]^. These findings suggest that miR-21 inhibitors - such as locked nucleic acid (LNA)-modified anti-microRNAs (anti-miRs), antagomirs, or small molecule modulators - could be employed to restore radiosensitivity.

Additionally, several other miRNAs, including miR-150, miR-483-5p, miR-454-3p, miR-31-5p, miR-613, and miR-340-3p, have been implicated in regulating angiogenesis and apoptosis in NPC^[69]^. Dysregulation of these miRNAs contributes to acquired radioresistance or, conversely, enhances radiosensitivity. Therapeutically, targeting these miRNAs using mimics or inhibitors could provide an avenue to modulate key signaling pathways, reduce angiogenesis, restore apoptotic responses, and improve RT outcomes. Overall, understanding the mechanistic roles of miRNAs in autophagy, angiogenesis, and apoptosis not only clarifies their contribution to radioresistance but also highlights opportunities for clinical intervention through miRNA-based therapies.

### Resistance to ICIs

ICs are immunosuppressive molecules that inhibit the immune system’s ability to recognize and eliminate cancer cells^[74]^. ICIs work by blocking these inhibitory pathways, restoring T-cell function. Commonly targeted pathways include programmed cell death protein 1 (PD-1)/programmed death-ligand 1 (PD-L1) and cytotoxic T-lymphocyte–associated antigen 4 (CTLA-4), along with other molecules such as lymphocyte activation gene 3 (LAG-3), and T-cell immunoglobulin and mucin domain-3 (TIM-3)^[75]^. ICIs have demonstrated improved survival outcomes across multiple cancers, including melanoma, lymphoma, esophageal carcinoma, and gastric cancer^[74]^. Despite these advances, a significant subset of patients exhibits primary or acquired resistance, underscoring the need for mechanistic understanding and predictive biomarkers.

In melanoma, resistance mechanisms often involve genetic alterations such as PTEN loss and impaired apoptotic signaling. MiRNAs, such as miR-4488, contribute to this resistance by promoting oncogenic signaling, migration, and invasion. Specifically, in melanoma, the MAPK pathway is a key regulator of tumor progression and is frequently targeted in combination with ICIs^[76]^. Another example is miR-4458, which functions as a tumor suppressor in various cancers, including hemangiomas, TNBC, HCC, and NSCLC. In NSCLC, decreased miR-4458 expression correlates with poor prognosis and reduced survival. Additionally, anti-PD-L1 immunotherapy further downregulates miR-4458 as NSCLC progresses. Functional studies using human NSCLC cell lines have shown that reduced miR-4458 expression promotes tumorigenicity. Wound healing assays revealed increased cell migration, Western blotting confirmed EMT marker expression, and the Edu assay demonstrated enhanced DNA synthesis, all indicative of increased tumor aggressiveness^[76]^. In parallel, overexpression of miR-155 has been shown to induce T-cell exhaustion through upregulation of PD-L1 on both tumor and myeloid cells, thereby impairing antitumor immunity. Conversely, inhibition of miR-155 restores T-cell cytotoxic activity and enhances responsiveness to ICIs^[77]^. The miR-34a family further exemplifies the therapeutic potential of miRNA modulation; downregulation of miR-34a in NSCLC and pancreatic cancer is associated with increased PD-L1 expression and diminished immune surveillance, whereas restoration of miR-34a reduces immune evasion and potentiates ICI efficacy^[78]^.

Other miRNAs, such as members of the miR-200 and let-7 families, regulate key pathways involved in resistance. Loss of miR-200c promotes EMT and elevates PD-L1 expression, facilitating immune escape and ICI resistance in lung and breast cancer models^[79]^. Similarly, let-7 downregulation in metastatic colorectal cancer, particularly in the context of KRAS mutations, diminishes therapeutic efficacy of cetuximab. Clinical studies reinforce the translational relevance of these observations. In one cohort of 234 patients treated with third-line cetuximab combined with irinotecan, overexpression of let-7 correlated with improved overall survival and increased responsiveness to therapy, demonstrating its potential as both a predictive biomarker and a therapeutic target^[80]^. These findings emphasize that genetic or epigenetic modulation of miRNA binding sites, including variants in the KRAS 3′-UTR, can influence drug sensitivity and provide opportunities to restore therapeutic responsiveness.

Integrating miRNA expression profiles with genomic and clinical data enhances the predictive power of patient stratification for ICI therapy. Machine learning approaches, such as random forest algorithms applied to miRNA datasets from metastatic melanoma patients, have successfully predicted treatment response and overall survival^[81]^. By combining tumor mutation burden, immune context, and miRNA signatures, these computational models achieve robust predictive accuracy, with validation metrics such as area under the curve (AUC) approaching 0.78^[82]^. Such integrative analyses not only highlight the mechanistic role of miRNAs in ICI resistance but also support their clinical utility as biomarkers for patient selection and as targets for novel adjuvant therapies. Modulating these miRNAs, either through mimics or inhibitors, offers a promising strategy to overcome resistance, enhance antitumor immunity, and improve the clinical efficacy of immunotherapy across diverse cancer types.

## MIRNAS AS BIOMARKERS FOR THERAPY RESISTANCE

Different cancer types exhibit distinct miRNA expression profiles, which are strongly associated with the tumor microenvironment and contribute significantly to therapeutic resistance. Resistance to chemotherapeutic agents such as cisplatin is influenced by miRNAs’ expression levels, which can either promote resistance or enhance sensitivity to treatment [Table 1]. Cisplatin-induced resistance is also linked to specific molecular pathways modulated by miRNAs, which alter cellular responses to the drug. Targeted therapies such as sorafenib and trastuzumab are similarly affected by miRNA regulation, as distinct miRNAs influence key signaling pathways, influencing drug efficacy and therapeutic outcomes [Table 2]. RT resistance is modulated in various cancers through persistent and repeated exposure to therapeutic doses, leading to the development of radioresistance, driven by adaptive cellular mechanisms [Table 3]. RT efficacy is also modulated by miRNA expression, which interacts with DNA repair and survival pathways to reduce treatment effectiveness. Lastly, the discovery of ICIs has provided a more targeted approach to inhibit ICs, suppressing natural immunity. To investigate the role of miRNAs in cancer treatment response, single-cell miRNA has been designed. In this workflow, synthetic 3′ and 5′ DNA adaptors are ligated to miRNAs, followed by polymerase chain reaction (PCR) amplification and next-generation sequencing. Validation in K562 cells demonstrated high reproducibility, with a coefficient of determination (R^2^) value of 0.930, confirming the robustness of the approach^[82]^. Furthermore, in a pilot study with seven small-cell lung cancer patients, analysis of circulating tumor cells (CTCs) revealed predominant expression of oncomiRs such as miR-21-5p, miR-146b-5p, miR-142-5p, miR-148a-3p, and miR-92a-3p, highlighting the potential of single-cell miRNA profiling for patient-specific biomarker discovery^[83]^. Furthermore, there has been data collected that incorporates circulating miRNAs as predictive biomarkers. For example, there was a clinical trial with advanced biliary tract cancer (ABTC) patients who received chemo-immunotherapy. The underlying issue is the lack of a reliable biomarker for predicting treatment response. The trial included a biliary tract cancer (BTC) focused plasma miRNA panel to identify miRNA expression. Therefore, three miRNAs, hsa-miR-16-5p, hsa-miR-93-5p, and hsa-miR-126-3p, were selected to validate the predictability of treatment response and overall survival. Each of the three demonstrated prognostic value regarding BTC^[84]^. These findings further align with the evidence that miRNAs have been found to regulate the efficacy of ICIs through modulation and regulation of each IC molecule [Table 4].

**Table 4:** MiRNAs’ role in resistance to cancer therapeutics - checkpoint inhibitors

**ncRNA**	**Cancer drug**	**Expression of miRNA**	**Cancer/cell type**	**Resistance pathway** **Resistance pathway**	**Ref.**
**Checkpoint inhibitors**					
Let-7 family, miR-199a, miR-375	Cetuximab (EGFR inhibitor)	Downregulated let-7 Upregulation of miR-199a and miR-375	Gastrointestinal cancer	KRAS, AKT	[59]
miR-4488	MAPK inhibitor	Downregulated	Melanoma	Expression of ZEB1 and CHK1	[85]
miR-155	Anti-PD-1	Downregulated	Melanoma	Overexpression of PTPN2	[85]
miR-125a	BRCA inhibitor	Upregulated	Melanoma	Intrinsic apoptotic pathway targeting BAK1 and MLK3	[86]
miR-4458	Anti-PD-L1	Downregulated	NSCLC	PD-L1 upregulated	[87]
miR-34a, miR-146a	Anti-PD-L1 and anti-CTLA-4	miR-34a upregulated miR-146a downregulated	HCC	Degradation of KLF4	[88]
Let-7b, Let-7a	Anti-CTLA-4	Downregulated	Head and neck squamous cell carcinoma	Targets regulation of TCF-4	[89]

MiRNAs: MicroRNAs; ncRNA: non-coding RNA; EGFR: epidermal growth factor receptor; KRAS: Kirsten rat sarcoma viral oncogene homolog; AKT: protein kinase B (PKB); MAPK: mitogen-activated protein kinase; ZEB1: zinc-finger E-box binding homeobox 1; CHK1: checkpoint kinase 1; PD-1: programmed cell death protein 1; PTPN2: protein tyrosine phosphatase non-receptor type 2; BRCA: BAK1: BCL2 antagonist/killer 1; MLK3: mixed lineage kinase 3; PD-L1: programmed cell death ligand 1; NSCLC: non-small cell lung cancer; CTLA-4: cytotoxic T-lymphocyte–associated antigen 4; HCC: hepatocellular carcinoma; KLF4: Krüppel-like factor 4; TCF-4: transcription factor 4.

Overall, various cancer treatments, including chemotherapy, targeted therapy, RT, and immunotherapy, are affected by miRNA-mediated mechanisms, which play a central role in drug resistance and treatment response across multiple cancer types.

## MIRNA-MEDIATED GENOMIC PROMOTERS OF DRUG RESISTANCE

MiRNAs, as key post-transcriptional regulators, are increasingly recognized for their involvement in cancer drug resistance, with their expression tightly influenced by genomic and genetic alterations. In epithelial ovarian cancer (EOC), which arises from the ovarian surface epithelium (OSE), high-throughput profiling of 18 validated EOC cell lines using TaqMan assays demonstrated that 160 out of 173 miRNAs were highly expressed, suggesting a global role of miRNAs in tumor biology^[90]^. Mechanistic studies in cell lines such as SKOV3 and OVCAR3/5 revealed that epigenetic silencing via DNA methylation and histone modifications contributes to the downregulation of tumor-suppressive miRNAs. Treatment with the DNA methylation inhibitor 5-aza-2′-deoxycytidine (5-Aza-CdR) and the histone deacetylase inhibitor 4-phenylbutyric acid (PBA) successfully restored miR-34b expression in six EOC cell lines, emphasizing the reversible nature of epigenetic regulation in miRNA-mediated tumor suppression^[90]^. Late-stage EOC frequently exhibits silencing of multiple tumor-suppressive miRNAs, correlating with altered mRNA transcript levels and poor patient survival.

Beyond epigenetic regulation, structural genomic changes such as copy number alterations (CNAs) also modulate miRNA expression. CNAs, defined as DNA duplications or deletions, can create dosage-sensitive effects on oncogenes and tumor suppressors, leaving a measurable genetic footprint relevant for therapeutic targeting^[91]^. Analysis of ovarian cancer samples from The Cancer Genome Atlas (TCGA) revealed that dosage-sensitive genes often coincide with miRNA regulatory networks. For instance, hsa-miR-100-5p and hsa-miR-99-5p were downregulated in dosage-sensitive RAD51C regions interacting with BRCA1/2 in DNA damage repair (DDR), whereas in dosage-resistant RAD51C samples, these miRNAs were upregulated, contributing to reduced expression of oncogenic genes and highlighting a context-dependent regulatory role^[91]^. These findings illustrate that integrating knowledge of CNAs, mutations, and miRNA expression can identify potential vulnerabilities that drive therapeutic resistance.

Predicting drug response and identifying therapeutic targets based on miRNA profiles alone can be challenging due to the complex, pleiotropic nature of miRNA regulation. Incorporating multi-omics datasets, including transcriptomic, genomic, and proteomic information, substantially improves the predictive power of miRNA-based analyses. For example, reverse-phase protein array (RPPA) data from breast cancer and ovarian cancer patients demonstrated a coordinated relationship between miRNAs and protein expression, including p27, MYC, and phospho-retinoblastoma (RB)^[92]^. Patients with low p27 and high MYC and phospho-RB levels were linked to dysregulated miRNAs, suggesting that miRNA networks can modulate key oncogenic proteins. Functional validation in MDA-MB-231 TNBC cells and ovarian cancer cell lines HeyA8 and SKOV3.ip1 identified a subset of 56 miRNAs capable of reversing the expression of these three critical proteins^[92]^. These observations underscore the potential of integrated multi-omics approaches to uncover mechanistic insights and improve therapeutic predictions. By combining miRNA profiles with genomic alterations (e.g., CNAs, mutations), transcriptomic patterns, and proteomic data, researchers can better stratify patients according to predicted drug responses and identify miRNAs that may serve as both biomarkers and therapeutic targets. Such integration enhances our ability to design precise, context-specific strategies to overcome drug resistance and improve clinical outcomes.

## THERAPEUTIC STRATEGIES TO TARGET MIRNAS TO OVERCOME THERAPY RESISTANCE

MiRNA mimics have recently been discovered as a potential therapeutic strategy to restore the function of downregulated tumor suppressor miRNAs in various cancers^[70]^. These mimics are synthetic oligonucleotides designed to replicate the function of endogenous miRNAs that are often lost or suppressed during tumor progression and therapeutic resistance. The inhibition process of tumor-suppressor miRNAs is depicted in Figure 1, highlighting the adverse effects of mature miRNAs on mRNA degradation and translation [Figure 1]. However, when external stimuli, such as miRNA mimic (drug influx), are introduced, they can restore these regulatory functions, leading to increased apoptosis and improved therapeutic resistance^[45]^. One therapeutic approach, known as miRNA replacement therapy, involves reintroducing or mimicking tumor suppressor miRNAs to inhibit oncogenic targets. This is conceptually similar to immunotherapy, which reactivates suppressed immune responses by targeting ICs. In miRNA replacement therapy, synthetic miRNAs bind and base-pair with complementary miRNA targets, promoting mRNA degradation or translation inhibition, thereby suppressing tumor-promoting genes^[41]^. In NSCLC, miR-181a expression is downregulated following cisplatin treatment in A549 cells. The use of a miR-181a mimic can restore expression, thereby enhancing antitumor responses and increasing sensitivity to cisplatin^[41]^. The previously mentioned miR-449 family - miR-449a, miR-449b-5p, and miR-449c-5p - exhibits similar tumor-suppressive functions^[85,86]^. In the TNBC cell line MDA-MB-231R, which lacks miR-449 expression, transfection with miR-449 mimics has been shown to restore miR-449 levels and reduce doxorubicin resistance^[87]^. These mimics act by downregulating the expression of cyclin-dependent kinase 2 (CDK2), E2F transcription factor 1 (E2F1), and E2F transcription factor 3 (E2F3), key regulators of cell cycle progression. This leads to cell cycle arrest, inhibiting DNA replication and division, and thereby enhancing sensitivity to doxorubicin^[87]^.

Complementing miRNA mimics, anti-miRs have been developed to inhibit overexpressed oncomiRs, such as miR-21. In human embryonic kidney (293T), NSCLC (A549), and metastatic lung cancer (NCI-H1299) cells, miR-21 and let-7a are highly expressed in A549 cells. Synthetic anti-miRNAs deplete these miRNAs, as validated using the miR-21-controlled dual reporter-expressing system (luc-21-miRDREL), where inhibition of miR-21 derepresses luciferase activity, allowing long-term monitoring of oncomiR activity in the tumor microenvironment. Anti-miRs thus restore tumor-suppressive gene expression and offer a precision strategy to overcome therapy resistance^[93]^. Interestingly, selective inhibition of tumor suppressor miRNAs can also have therapeutic benefits in certain contexts. Hsa-miR-148a-3p, normally downregulated in cancers such as breast cancer, was directly inhibited using cross-linked antisense oligonucleotides (CL-AMO). Transfection with CL-miR-148a suppressed miR-148a levels in a dose-dependent manner over nine days, leading to reduced proliferation in MCF-7 cells via upregulation of thioredoxin interacting protein (TXNIP)^[94]^. This demonstrates the dual utility of miRNA therapeutics, depending on their oncogenic or tumor-suppressive context.

Advances in delivery technologies have markedly improved the precision and efficacy of miRNA-based therapies. Nanoparticles, for instance, protect miRNA mimics or inhibitors from degradation during systemic circulation and enhance tumor-specific delivery. Vyxeos, a U.S. Food and Drug Administration (FDA)-approved liposomal formulation co-delivering cytarabine and daunorubicin for AML, exemplifies how nanoparticle delivery improves drug stability and targets proliferative bone marrow niches^[95]^. Similarly, lipid nanoparticles (LNPs) encapsulating oncosuppressor miRNAs, such as miR-204-5p and miR-199b-5p, have been employed in preclinical models of BRAF-mutant melanoma to enhance the efficacy of MAPK inhibitors. In A357 and M14-derived tumors, LNP-miRs reduced tumor growth, increased miRNA expression *in situ*, and decreased phosphorylated ERK (pERK) levels, demonstrating synergistic tumor suppression^[96]^. Beyond nanoparticles, viral vector-based delivery represents an additional avenue for miRNA therapeutics. Adeno-associated viruses (AAVs) and lentiviral systems allow stable expression of miRNA mimics or sponges in tumor cells, ensuring prolonged activity *in vivo* while minimizing off-target effects. Combined with chemotherapeutics or targeted therapies, these strategies offer a versatile platform for reversing drug resistance, restoring tumor-suppressive pathways, and enhancing anti-cancer efficacy. Collectively, these approaches - miRNA mimics, anti-miRs, nanoparticle-based systems, and viral vectors - provide a comprehensive toolkit to modulate miRNA activity and overcome therapy resistance in diverse cancer contexts.

## FUTURE PERSPECTIVES

Over the past decades, substantial progress in cancer therapy has yielded increasingly precise and effective treatment modalities. Alongside these clinical advancements, important discoveries have emerged regarding the molecular and cellular mechanisms regulating therapy sensitivity. Factors such as the tumor microenvironment, inherent resistance mechanisms, and miRNA-mediated regulation critically shape treatment outcomes. Despite these improvements, the nonspecific nature of many conventional therapies continues to result in off-target effects, highlighting the need for more precise and personalized interventions.

A deeper understanding of how specific therapies interact with molecular regulators is essential to overcome resistance and improve outcomes. For instance, while targeted therapies and ICIs can effectively reduce tumor burden, they may also induce cellular plasticity or compensatory pathways that contribute to acquired resistance. Therapeutic strategies increasingly focus on modulating miRNA networks to restore tumor-suppressive functions or inhibit oncomiRs, thereby enhancing sensitivity to chemotherapy, RT, and immunotherapy. For example, dual checkpoint blockade combining PD-1/PD-L1 inhibitors with CTLA-4 inhibitors has demonstrated synergistic enhancement of T cell-mediated antitumor immunity, and additional approaches targeting immunosuppressive cytokines, such as transforming growth factor beta (TGF-β) or interleukin 10 (IL-10), are under investigation to remodel the tumor microenvironment and overcome immune resistance^[97]^. Similarly, nanoparticle-mediated delivery of miRNA mimics or anti-miRs offers a strategy to fine-tune gene expression networks that drive therapy resistance, enabling combinatorial approaches with conventional chemotherapeutics or targeted inhibitors. Viral vector-based delivery of miRNA therapeutics is also emerging as a promising approach, allowing sustained and cell-specific modulation of miRNA activity. Emerging single-cell technologies are poised to transform our understanding of miRNA heterogeneity within tumors. Single-cell miRNA sequencing, for example, allows parallel profiling of miRNA and mRNA expression in individual cells through half-cell genomics, in which a single cell is lysed and split into two fractions for simultaneous miRNA and mRNA analysis. Advances such as chemically modified adapters, exonuclease digestion of excess adapters, and CleanTag adapters further enhance the sensitivity and specificity of these assays, reducing adapter-dimer formation and improving library quality^[83]^. Integrating single-cell miRNA sequencing with genomic, transcriptomic, and proteomic datasets represents a concrete future direction to address the current limitations of open-access miRNA datasets and improve predictive power for therapy response. For example, combining miRNA profiles with CNAs, mutation status, and protein expression can identify dosage-sensitive genes and uncover miRNA-driven regulatory networks that contribute to drug resistance. In ovarian cancer, miRNAs such as hsa-miR-100-5p and hsa-miR-99-5p were shown to regulate RAD51C expression, a key component of DDR, suggesting that miRNA-based biomarkers could predict therapeutic outcomes and guide personalized treatment decisions^[98]^. Similarly, proteogenomic analyses in breast and ovarian cancer demonstrated that subsets of 56 miRNAs could potentially reverse aberrant expression of oncogenic proteins such as MYC, phospho-RB, and p27, offering a framework for combinatorial therapeutic interventions^[92]^.

In conclusion, future directions in cancer therapy will increasingly leverage miRNA modulation as both diagnostic biomarkers and therapeutic targets. Integration of high-resolution single-cell profiling, multi-omics datasets, and advanced delivery systems - including nanoparticles and viral vectors - offers a path to overcome therapy resistance, enhance precision medicine, and develop patient-specific treatment strategies. These advances promise to narrow the current gaps in miRNA-based datasets, facilitate mechanistic insights, and translate into more effective clinical interventions.

## DECLARATIONS

### Copyright

© The Author(s) 2025.
